# Genome‐resolved metagenomics of a bioremediation system for degradation of thiocyanate in mine water containing suspended solid tailings

**DOI:** 10.1002/mbo3.446

**Published:** 2017-02-19

**Authors:** Sumayah F. Rahman, Rose S. Kantor, Robert Huddy, Brian C. Thomas, Andries W. van Zyl, Susan T.L. Harrison, Jillian F. Banfield

**Affiliations:** ^1^ Department of Plant and Microbial BiologyUniversity of CaliforniaBerkeleyCAUSA; ^2^ Department of Chemical EngineeringCenter for Bioprocess Engineering ResearchUniversity of Cape TownCape TownSouth Africa; ^3^ Department of Earth and Planetary SciencesUniversity of CaliforniaBerkeleyCAUSA; ^4^ Department of Environmental Science, Policy, and ManagementUniversity of CaliforniaBerkeleyCAUSA

**Keywords:** biodegradation, bioreactors, metabolic pathways, metagenomics, thiocyanate

## Abstract

Thiocyanate (SCN^−^) is a toxic compound that forms when cyanide (CN^−^), used to recover gold, reacts with sulfur species. SCN^−^‐degrading microbial communities have been studied, using bioreactors fed synthetic wastewater. The inclusion of suspended solids in the form of mineral tailings, during the development of the acclimatized microbial consortium, led to the selection of an active planktonic microbial community. Preliminary analysis of the community composition revealed reduced microbial diversity relative to the laboratory‐based reactors operated without suspended solids. Despite minor upsets during the acclimation period, the SCN^−^ degradation performance was largely unchanged under stable operating conditions. Here, we characterized the microbial community in the SCN^−^ degrading bioreactor that included solid particulate tailings and determined how it differed from the biofilm‐based communities in solids‐free reactor systems inoculated from the same source. Genome‐based analysis revealed that the presence of solids decreased microbial diversity, selected for different strains, suppressed growth of thiobacilli inferred to be primarily responsible for SCN^−^ degradation, and promoted growth of *Trupera*, an organism not detected in the reactors without solids. In the solids reactor community, heterotrophy and aerobic respiration represent the dominant metabolisms. Many organisms have genes for denitrification and sulfur oxidation, but only one *Thiobacillus* sp. in the solids reactor has SCN^−^ degradation genes. The presence of the solids prevented floc and biofilm formation, leading to the observed reduced microbial diversity. Collectively the presence of the solids and lack of biofilm community may result in a process with reduced resilience to process perturbations, including fluctuations in the influent composition and pH. The results from this investigation have provided novel insights into the community composition of this industrially relevant community, giving potential for improved process control and operation through ongoing process monitoring.

## 1 INTRODUCTION

Cyanide (CN^−^) is used globally in the gold mining industry as a lixiviant to dissolve and remove gold from ore. During gold extraction by cyanidation, CN^−^ can react with reduced sulfur species, forming thiocyanate (SCN^−^) in the gold mining effluents. Although SCN^−^ is not as toxic as CN^−^, it is known to be harmful to humans and aquatic organisms (Boening & Chew, 1999; Erdogan, 2003; Shifrin, Beck, Gauthier, Chapnick, & Goodman, 1996), requiring the use of chemical or biological methods for its removal. The use of microbes for biological remediation of SCN^−^ from contaminated wastewater has been successful at both the laboratory scale (Boucabeille, Bories, Ollivier, & Michel, 1994; du Plessis, Barnard, Muhlbauer, & Naldrett, 2001; van Zyl, Harrison, & van Hille, 2011; van Zyl, Huddy, Harrison, & van Hille, 2015) and in commercial operations (van Buuren, Makhotla, & Olivier, 2011). Engineers have developed and commercialized a SCN^−^ biodegradation process known as Activated Sludge Tailings Effluent Remediation (ASTER^™^) that involves continuous feeding of SCN^−^‐containing solutions into aerated bioreactors to promote microbial degradation (van Buuren et al., 2011).

Two metabolic pathways have been proposed for the biological degradation of SCN^−^. In one pathway, thiocyanate hydrolase converts SCN^−^ to sulfide and cyanate (OCN^−^). OCN^−^ is further hydrolyzed to carbon dioxide and ammonium, while sulfide is oxidized to sulfate. In the other degradation pathway, SCN^−^ is hydrolyzed into carbonyl sulfide (OCS) and ammonium. OCS can be broken down into carbon monoxide and sulfide, which is then oxidized to sulfate (Katayama et al., 1992; Katayama et al., 1998).

To identify the microorganisms responsible for SCN^−^ degradation, microbial communities in experimental reactors have been characterized by molecular fingerprinting (Felföldi et al., 2010; Huddy, van Zyl, van Hille, & Harrison, 2015; Quan et al., 2006) and genome‐resolved metagenomic analysis (Kantor et al., 2015; R. S. Kantor, R. J. Huddy, I. Ramsunder, B. C. Thomas, S. Tringe, R. L., Hettich, S. T. L., Harrison, J. F. Banfield, in review). Analysis of the 16S and 18S rRNA in a reactor established with an ASTER^™^ consortium revealed that the microbial community was much more diverse than previously expected (Huddy et al., 2015). Metagenomic analysis of the same system predicted the metabolic potential of the key organisms (e.g., *Thiobacillus* spp.) and described the potential flow of carbon, sulfur, and nitrogen through the community (Kantor et al., 2015).

In the laboratory‐based SCN^−^‐degrading system described by previous studies, SCN^−^‐containing synthetic wastewater was fed to the laboratory reactors and, where the SCN^−^ feed concentration was sufficiently high, thick biofilms formed on all reactor surfaces. Biofilm improves SCN^−^ degradation rates, in part by ensuring biomass retention during continuous flow mode and by enhancing process robustness for dynamic waste streams (Huddy et al., 2015). Typically, the ASTER^™^ process is not performed in the presence of particulate tailings (i.e., mineral particles left behind after separating the gold from ore concentrate). However, at some mining sites, the removal of solid tailings from the effluent is not achieved fully due to site topography, particle size, density of the tailings, and other factors (van Zyl et al., 2015). In a bioreactor inoculated with the microbial consortium of the SCN^−^ stock reactor (Kantor et al., 2015), van Zyl et al. (2015) acclimatized the microbial community to an incrementally increasing loading of solids of density 2.7 g/L to a final concentration of 5.5% m/v, and showed that, following acclimatization, SCN^−^ degradation still occurred. However, biofilm did not form on the submerged surfaces of the reactor. Following an extended period of continuous operation, this solids‐containing reactor was operated in “draw and fill” mode, meaning that fluid was removed periodically and the volume replaced with untreated fluid.

This study was motivated by the use of the acclimatized microbial culture, as developed by van Zyl et al. (2015), as the inoculum for an ASTER^™^ process to treat the effluent from a bioleaching operation exploiting a refractory gold deposit in the Philippines. The aim of the research was to resolve the microbial community associated with an active ASTER^™^ solids reactor system and to compare that with previously resolved (Kantor et al., 2015; R. S. Kantor, R. J. Huddy, I. Ramsunder, B. C. Thomas, S. Tringe, R. L., Hettich, S. T. L., Harrison, J. F. Banfield, in review) ASTER^™^ microbial communities. In this study, we used genome‐resolved metagenomics to elucidate the microbial community composition and metabolic potential of the solids‐containing SCN^−^ degradation bioreactor. We hypothesized that due to a lack of biofilm in the solids reactor (van Zyl et al., 2015), there would be differences in community membership compared to the reactors without solids. Moreover, we hypothesized that given the lower SCN^−^ loading in this system, key SCN^−^ degrading organisms may be at lower relative abundances in this reactor compared to solids‐free reactors at higher loading rates. Here, we report the composition and metabolic potential of the solids reactor microbial community.

## 2 MATERIALS AND METHODS

### 2.1 Study samples

#### 2.1.1 Mineral solids

The mineral solids were generated by SGS (Johannesburg) and provided by Gold Fields, as described by van Zyl et al. (2015). The fine‐grained particulates had a D_50_ of 6.122 μm (D_10_ of 0.939 μm and D_90_ of 38.026 μm) and a density of 2.677 g/ml.

#### 2.1.2 The ASTER^™^ culture

The mixed microbial consortium used to inoculate the reactors was derived from the stock ASTER^™^ culture, with prior characterization reported by Huddy et al. (2015) and Kantor et al. (2015). It was acclimatized to cultivation in the presence of suspended solids as described by van Zyl et al. (2015).

#### 2.1.3 Reactor system

The work was conducted using a stirred tank reactor, with an operating volume of 1 L, as described by van Zyl et al. (2015). The microbial culture, acclimatized during the investigation by van Zyl et al. (2015), was maintained in a “draw‐and‐fill” culture with a 10% volume replacement by a feed solution, containing the solids (5.5% m/v), SCN^−^ (450 mg/L, as KSCN), molasses (150 mg/L) and phosphate (27 mg/L, as KH_2_PO_4_) on a weekly basis. The molasses was provided to support heterotrophic growth. The pH of the feed was initially adjusted, using potassium hydroxide to maintain the reactor at approximately pH 7.0.

### 2.2 DNA extraction and sequencing

Two separate samples of approximately 15 ml were drawn from the well‐mixed suspended solids reactor operated under the same conditions at an interval of 25 days. These samples were processed independently. The biomass was harvested by centrifugation (14,000 rpm for 10 min at 22°C). Total DNA was extracted using a NucleoSpin^®^ soil genomic DNA extraction kit (Machery‐Nagel, Germany) with the inclusion of a repeated extraction step, according to the manufacturer's instructions. Paired end library preparation and sequencing were performed with Illumina HiSeq 2,500 run at the rapid mode at the Joint Genome Institute (Walnut Creek, CA). An insert size of 500 bp was used to yield 251 bp reads.

### 2.3 Read processing, assembly, and initial functional annotation

For both datasets, reads were hard trimmed to 150 bp and processed by BBtools to remove Illumina adapters and trace contaminants. The reads were then trimmed for quality, using Sickle with default settings (https://github.com/najoshi/sickle). The datasets were assembled independently, using idba_ud with the pre‐correction option, for normalization of highly represented kmers (Peng, Leung, Yiu, & Chin, 2012). Genes on scaffolds ≥ 1,000 bp were predicted, using Prodigal with the metagenome option (Hyatt, Locascio, Hauser, & Uberbacher, 2012). For annotation, similarity searches were performed, using USEARCH, which compares sequences against the KEGG, UniRef100, and UniProt databases. KEGG and UniRef100 were searched in the forward and reverse direction to identify reciprocal best hits, while only forward searches were done for UniProt. The phylogenetic affiliation to the lowest possible taxonomic level was determined based on the best hit against the UniRef100 database; 16S rRNA genes were predicted based on the ssu‐align‐0p1.1.cm database, and transfer RNA genes were predicted, using tRNAscanSE (Lowe & Eddy, 1997).

### 2.4 Genome binning and dereplication

Genome bins were assigned based on coverage, GC content, and the phylogenetic best‐hit profile of scaffolds ≥ 1,000 bp, using ggkbase binning tools (ggkbase.berkeley.edu). Emergent‐self organizing maps (ESOMs) based on di‐ and tri‐nucleotide frequencies and differential coverage across the samples were created for each of the two datasets (Dick et al., 2009), and the tentative bin information was superimposed onto the ESOMs as class files, using the Databionic ESOM Tool, esomana (Ultsch & Moerchen, 2005). The bins were checked manually, and any mis‐binned scaffolds were transferred to the correct bin. The bacterial genomes were curated to resolve assembly errors, extend scaffolds, and join scaffolds. Genome completeness for the bacterial bins was assessed based on the presence or absence of 51 bacterial single copy genes that are widely conserved. The genome bins from the solids 1 and solids 2 samples were aligned, and bins with >98% nucleotide identity across 50% of the genome were classified as the same genome. The winning genome was chosen based on genome completeness and included in the dereplicated solids dataset. To determine which organisms from the solids bioreactor have been found previously in thiocyanate bioreactors, genomes were clustered at >98% average nucleotide identity, using the MinHash technique (Ondov et al., 2016). Read mapping for coverage calculation was performed, using Bowtie2 with default settings (Langmead & Salzberg, 2012). If an organism occurred with coverage >1×, it was considered to be present in that sample.

### 2.5 Phylogenetic analysis based on ribosomal protein sequences

The genes for 16 ribosomal proteins (L2, L3, L4, L5, L6, L14, L16, L18, L22, L24, S3, S8, S10, S17, and S19) were collected from the solids 1 and solids 2 datasets, as well as the SCN‐only (SCN^−^ loading rate of 1.9 mmol L^−1^ hr^−1^; 12 hr residence time; Kantor et al., 2015), CN–SCN (SCN^−^ and CN^−^ loading rate of 0.9 and 0.14 mmol L^−1^ hr^−1^, respectively, at a 14 hr residence time; Kantor et al., 2015), and SCN^−^ two‐reactor time series datasets (SCN^−^ loading rate of 0.07–1.4 mmol L^−1^ hr^−1^ at a 12 hr residence time and SCN^−^ feed concentrations from 50 to 1,000 mg/L; R. S. Kantor, R. J. Huddy, I. Ramsunder, B. C. Thomas, S. Tringe, R. L., Hettich, S. T. L., Harrison, J. F. Banfield, in review), excluding those from bins labeled as eukaryotes, viruses, phage, plasmids, or mitochondria. These 16 genes, along with the 16 ribosomal protein genes from a custom reference set, were aligned independently with MUSCLE (Edgar, 2004). The alignments were trimmed to remove ambiguously aligned termini and columns composed of more than 95% gaps. The alignments were then concatenated to form an alignment with 2,454 columns, and taxa that had less than 50% of the alignment were removed. Due to incomplete sequences, some organisms from the datasets did not get incorporated into the final concatenated alignment. This alignment was used to construct a maximum likelihood phylogeny with RAxML, using the PROTGAMMALG model (Stamatakis, 2014).

### 2.6 Metabolic analysis

Genome‐specific metabolic potential was determined by the following: (1) searching all predicted ORFs in a genome with Pfam30, TIGRfam31, Panther32 and custom HMM profiles of marker genes for specific pathways, using hmmscan33 (Anantharaman et al., 2016); (2) assessment of metabolic pathways using annotations on ggKbase (ggkbase.berkeley.edu); and (3) searching particular proteins of interest, using BLAST (Altschul, Gish, Miller, Myers, & Lipman, 1990). For a generation of custom HMM profiles, references for each marker gene were aligned using MUSCLE and the start and ends of the alignment were manually trimmed. The alignment was converted into Stockholm format and databases were built, using hmmscan33. For RuBisCO and hydrogenases34, different hmm databases were constructed for each distinct group. Individual cutoffs for all HMMs were determined by manual inspection. To compare genomes found in thiocyanate bioreactors with published genomes, the genomes of interest were downloaded from NCBI, and reciprocal BLASTs were utilized to identify shared and unique genes.

## 3 RESULTS AND DISCUSSION

### 3.1 Genome recovery and community structure

Metagenomic sequencing for two samples from the SCN^−^ bioreactor with solids was obtained: the “solids 1” sample (4.6 Gbp of sequence) was taken 1 month prior to “solids 2” (5.1 Gbp of sequence). Raw read data for solids 1 and solids 2 can be accessed at NCBI with accession numbers SAMN05509838 and SAMN05509839. *De novo* assembly of the metagenomes resulted in a 178.9 Mbp assembly for solids 1 (with 51% of the assembly in contigs ≥ 5 kb) and a 213.2 Mbp assembly for solids 2 (with 77% in contigs ≥ 5 kb). Genome binning based on GC content, coverage, di‐and tri‐nucleotide frequencies, and differential coverage across the solids 1 and solids 2 samples yielded 34 bacterial genomes from solids 1 and 25 bacterial genomes from solids 2. The taxonomic compositions of the two samples were similar. However, in solids 1, we also reconstructed draft mitochondrial genomes for two protozoa and a partial genome of yeast belonging to the Saccharomycetales.

**Figure 1:**
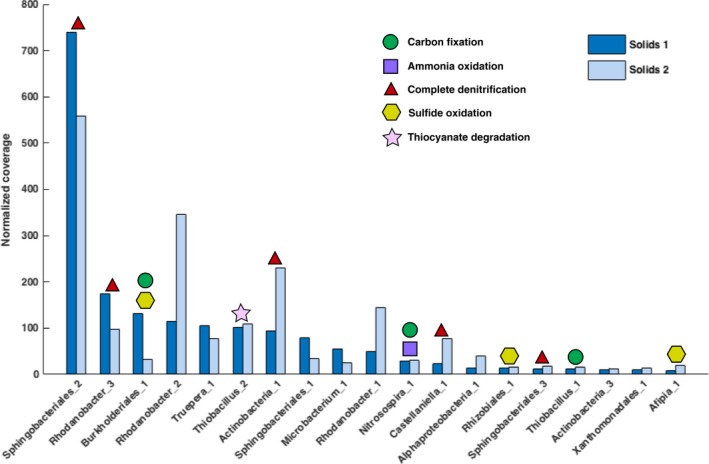
Metabolic potential of the 19 organisms present at a high enough abundance in both the solids 1 and solids 2 samples to allow for genome‐based analysis. The number of raw reads for each sample was used to normalize the coverage data, in order to accurately compare the two samples

**Figure 2:**
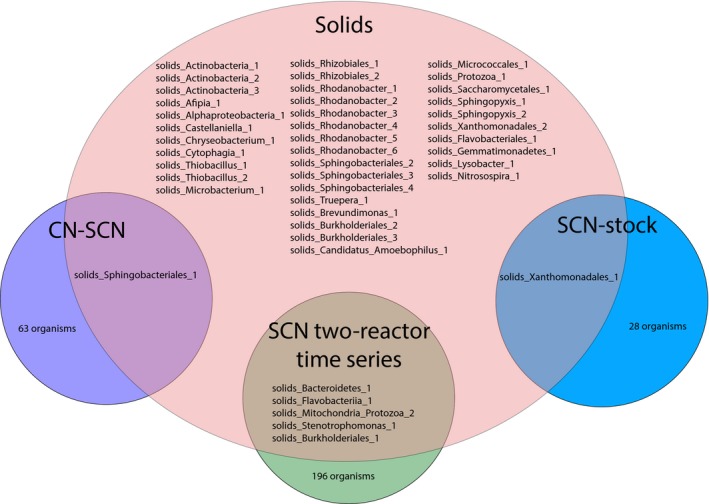
Illustration of the overlaps among reactor communities. To identify overlapping genomes, representative parts of the genome bins were aligned and clustered based on >98% average nucleotide identity

Based on coverage data, Sphingobacteriales_2 was consistently the dominant organism and *Thiobacillus* spp. were present at only moderate abundance (Figure 1), unlike in SCN^−^ bioreactors without solids where thiobacilli were the most abundant community members (Kantor et al., 2015). Phylogenetic analysis (Figure S1) revealed that the two thiobacilli in the solids reactor are closely related to, but distinct from, the strains reported from solids‐free reactors with the same inoculum (Figure 2).

To identify overlapping genomes in solids 1 and solids 2, genomes with >98% nucleotide identity were clustered. This resulted in a dereplicated dataset of 40 bacterial genomes, available at http://ggkbase.berkeley.edu/scnpilot_solids_dereplicated/organisms. Out of the 34 distinct bacterial genomes in solids 1 that were abundant enough for at least partial genome‐based analysis (>.25% of the community), 19 were also sufficiently abundant for genome‐based analysis in solids 2 (Figure 1). The relative abundances of some of these organisms did not change between time points (e.g., Truepera_1), whereas others decreased (e.g., Sphingobacteriales_2) or increased dramatically (e.g., Rhodanobacter_2) (Figure 1). However, based on an analysis involving stringent read mapping that enabled detection of organisms at relative abundance levels of ~.06% of the community, 39 out of the 40 unique bacterial genomes were detected in both samples (Table S1).

### 3.2 Dominance of heterotrophy and aerobic metabolism

Metabolic analyses revealed that no genomes from the solids bioreactor possess the genes for the Wood–Ljungdahl pathway or the reverse tricarboxylic acid (TCA) cycle. Four genomes carry genes for form I and/or form II RuBisCO (*rbc*), the key enzyme of the Calvin–Benson–Bassham cycle (Table S1). The lack of carbon fixation genes in 90% of genomes from this dataset indicates that the community was mainly composed of heterotrophs. In fact, only one of the five most abundant (and genomically well‐defined) organisms in the solids reactor is an autotroph (Figure 1). Heterotrophs likely consume the molasses in the media as well as biomass and/or organic exudates or lysis products from autotrophs. In contrast, three of the five most abundant organisms in the solids‐free bioreactor are autotrophs (Kantor et al., 2015).

To determine the oxygen requirements for organisms in the solids bioreactor, we searched the genome bins for the presence of cytochrome oxidase genes. The vast majority of genomes contain at least one cytochrome oxidase, indicating the ability to use oxygen as a terminal electron acceptor. Just one near‐complete genome, the predicted endosymbiont Cytophagia_1, lacks cytochrome oxidase (Table S1). The presence of most of the glycolysis pathway, in addition to pyruvate dehydrogenase, suggests that Cytophagia_1 may ferment pyruvate, possibly producing acetate as a metabolic byproduct. The dominance of aerobic organisms is not surprising, given that the solids reactor is well aerated and does not develop biofilm (van Zyl et al., 2015), which would provide anaerobic and microaerobic environments (Falsetta, McEwan, Jennings, & Apicella, 2010; Fox et al., 2014).

### 3.3 Thiocyanate, nitrogen and sulfur compound metabolic pathways

Thiobacillus_2, the more abundant of the two identified *Thiobacillus* strains (Figure 1), possesses a thiocyanate hydrolase (*scnABC*) (Table S1), the enzyme involved in the degradation of SCN^−^ in *Thiobacillus thioparus* THI115 (Arakawa et al., 2007; Kataoka et al., 2006), and the genes for this enzyme are located in a conserved operon as previously described (Kantor et al., 2015). Both Thiobacillus_1 and Thiobacillus_2 possess genes involved in sulfur oxidation and denitrification (Table S1). Thus, it is clear that *Thiobacillus* spp. have important roles in the solids reactor, although they are not the dominant organisms (Figure 1) as they were in the SCN^−^ stock reactor (Kantor et al., 2015). The decrease in the proportion of *Thiobacillus* in the solids bioreactor relative to the reactors operated without solids and the reduced number of species with SCN^−^ degradation ability may explain the increased sensitivity of the SCN^−^ degradation to process perturbation and stress as reported by van Zyl et al. (2015).

SCN^−^ degradation results in the production of ammonium that could be converted into nitrite and removed by denitrification. Only one genome bin, Nitrosospira_1, contains genes for ammonium oxidation, *amo* and *hao* (Table S1), suggesting that this organism is critical for nitrite production in the system. We detected no genes for anaerobic ammonium oxidation in the dataset, as was the case in studies of solids‐free reactors (Kantor et al., 2015; R. S. Kantor, R. J. Huddy, I. Ramsunder, B. C. Thomas, S. Tringe, R. L., Hettich, S. T. L., Harrison, J. F. Banfield, in review). Six organisms in the solids reactor contain a full denitrification pathway (including *nar*,* nir*,* nor*, and *nos* genes) for the complete reduction of nitrate to N_2_ (Table S1). Other genomes were missing one or more genes in the denitrification pathway, although this may be due to incomplete genome recovery. The dominant organism, Sphingobacteriales_2, is likely the main contributor to denitrification in the system (Figure 1).

An important step in the SCN^−^ breakdown pathway is sulfur oxidation. For the oxidation of sulfide, either SoxCD or rDsrAB is required. The gene for SoxC, which forms a complex with SoxD and works in conjunction with the other Sox enzymes, is present in four genomes (Table S1). Thiobacillus_1 contains *dsrAB,* which may function in the reverse dissimilatory sulfite reductase pathway that can oxidize sulfur to sulfite. We identified genes for adenosine phosphosulfate reductase (*apr*) in Thiobacillus_2 and adenosine triphosphate sulfurylase (*atpS*) in Xanthomonadales_1; these may complete the oxidation by converting sulfite to sulfate. Other genes known to be involved in the oxidation of sulfur compounds, such as *fcc* and *sqr*, were found in several genomes in this dataset (Table S1). Overall, we conclude that based on its high abundance, Burkholderiales_1 is the most important organism involved in sulfur compound oxidation, although Rhizobiales_1 and Afipia_1 likely also contribute to these reactions (Figure 1).

### 3.4 An organism in the solids reactor not found in the solids‐free reactors

A bacterium of the phylum Deinococcus–Thermus occurred in both the solids 1 and solids 2 samples (Figure 1). To our knowledge, this is the first reporting of a Deinococcus–Thermus in bioreactors inoculated with the ASTER^™^ microbial consortium (du Plessis et al., 2001; Huddy et al., 2015; Kantor et al., 2015; R. S. Kantor, R. J. Huddy, I. Ramsunder, B. C. Thomas, S. Tringe, R. L., Hettich, S. T. L., Harrison, J. F. Banfield, in review; van Buuren et al., 2011; van Zyl et al., 2015). We reconstructed a draft Truepera_1 genome that is 1.22 Mbp in length with 90% of expected single copy genes (Table S1). In comparison, the published complete genome of *Truepera radiovictrix*, the only genome available from the *Truepera* genus, is 3.23 Mbp in length (Ivanova et al., 2011). The 16S rRNA gene of *T. radiovictrix* shares only 89% identity with the sequence from Truepera_1, so it is possible that the two organisms do not belong to the same Genus; however, *T. radiovictrix* is the nearest sequenced relative. Members of Deinococcus–Thermus are known to be highly resistant to environmental hazards; specifically, *T. radiovictrix* is resistant to ionizing radiation and can grow under extreme conditions such as high alkalinity (Albuquerque et al., 2005). Given that *T. radiovictrix* is an alkaliphile, it was surprising that Truepera_1 was not also detected in the solids‐free reactor, which has a higher pH than the solids reactor (Huddy et al., 2015; van Zyl et al., 2015).

We compared the newly reconstructed Truepera_1 genome to that of *T. radiovictrix*, as it is the closest reference available. Like the published *T. radiovictrix* strain RQ‐24^T^ *,* Truepera_1 is predicted to be an aerobic heterotroph. Unlike the reference sequence, Truepera_1 has genes for the export of heavy metals. There were also several genes present in the published *Truepera* genome that are not in Truepera_1, although this may be due to the fact that the Truepera_1 genome is incomplete. These included genes for L‐lactate dehydrogenase, which *T. radiovictrix* strain RQ‐24^T^ utilizes when it switches to homolactic fermentation, and manganese catalase, an antioxidant defense metalloenzyme that may be involved in strain RQ‐24^T^'s resistance to ionizing radiation.

Truepera_1 thrives in the well‐aerated solids bioreactor as the fifth most abundant organism (Figure 1), where it most likely consumes the molasses in the reactor feed. As the genome harbors a copper‐containing nitrite reductase NirK, Truepera_1 may play a role in the denitrification process within the bioreactor (Table S1). The differing conditions in the solids reactor, including the SCN^−^ loading rate, likely resulted in the enrichment of low‐abundance organisms that were not detected previously, such as Truepera_1. A notable feature of the solids bioreactor is that the high agitation of solid tailings causes shear stress that prevents biofilm formation (Illing & Harrison, 1999; van Zyl et al., 2015). Mechanisms for resistance to shear stress have been identified in other members of the Deinococcus–Thermus; for example, the SlpA protein in *Deinococcus radiodurans* R1 maintains cell envelope integrity (Rothfuss, Lara, Schmid, & Lidstrom, 2006). One S‐layer protein gene was found in the Truepera_1 genome, and its best hit in the NCBI Protein database is the S‐layer protein of *D. radiodurans* R1. If Truepera_1 has capabilities similar to *D. radiodurans* that allow it to resist the shear stress brought about by the agitated solids, this may contribute to its proliferation in this bioreactor.

### 3.5 Viruses and eukaryotes

Viruses and phage were abundant in the solids reactor. Two eukaryotic viruses and twenty phages were binned from the dataset, with five of the phage occurring in both the solids 1 and solids 2 samples. Some phage genomes were found within the genome bins of specific bacteria based on co‐abundance patterns, suggesting possible affiliations. These bacteria include Rhizobiales_1, Rhizobiales_2, Xanthomonadales_1, Burkholderiales_1, Afipia_1, Chryseobacterium_1, and Rhodanobacter_2. A virus was found in the eukaryotic genome bin Saccharomycetales_1. These findings may indicate that viruses and phage play important roles in carbon turnover in the bioreactor. Metagenomic analysis of the bioreactors without solids also suggested that predation by eukaryotes and phage affects community dynamics (Kantor et al., 2015).

The genome for the yeast Saccharomycetales_1 was 8.52 Mbp in length and appears to be around half‐complete, given 690 complete single‐copy Benchmarking Universal Single‐Copy Orthologs (BUSCOs) out of 1,438 total BUSCO groups searched (Simão, Waterhouse, Ioannidis, Kriventseva, & Zdobnov, 2015). Other organisms belonging to the order Saccharomycetales have been previously found in this system. An analysis of 18S rRNA genes from the solids bioreactor revealed the presence of a yeast that is a close relative of *Candida palmioleophila* (van Zyl et al., 2015), and the ASTER^™^ microbial consortium has been found to include *Candida humulis* (van Buuren et al., 2011). In a study that utilized light microscopy, yeast‐like cells and other eukaryotes such as filamentous fungi were present in the biofilm of the SCN^−^ reactor without solids (Huddy et al., 2015). The presence of Saccharomycetales_1 in the solids bioreactor, which does not contain biofilm, indicates that yeasts can also occur in the liquid portion of the bioreactor.

Mitochondrial genomes for two protozoa were identified in the solids 1 dataset. Mitochondria_Protozoa_1 was classified as *Acanthamoeba castellanii*, a unicellular amoeba that frequently captures prey by phagocytosis and harbors bacterial endosymbionts (Khan, 2001). In the solids reactor, Protozoa_1 likely carried the bacterial symbiont Cytophagia_1 based on co‐abundance patterns. Mitochondria_Protozoa_2, which was also observed in one of the solids‐free bioreactors (Figure 2), was classified as a Schizopyrenida. The majority of the contigs within the Mitochondria_Protozoa_2 genome bin corresponded to *Naegleria*, which are organisms known for their ability to transform from an amoeba to a flagellate (Marciano‐Cabral, 1988).

An interesting phenomenon is the lack of rotifers in the solids reactor, which have been identified and observed in bioreactors without solids (R. S. Kantor, R. J. Huddy, I. Ramsunder, B. C. Thomas, S. Tringe, R. L., Hettich, S. T. L., Harrison, J. F. Banfield, in review). The rotifers prefer the planktonic portion of these reactors and feed on the edges of the biofilm. The shear stress caused by the highly agitated tailings material in the solids bioreactor may hinder the survival of these pseudocoelomate animals. Additionally, the slightly acidic conditions in the solids reactor (van Zyl et al., 2015) may not be ideal for these planktonic rotifers (Bērziņš & Pejler, 1987).

## 4 CONCLUSIONS

Bacteria from seven phyla were detected in the solids bioreactor (present at >.06% of the community) (Table S1). In contrast, a sample from the SCN^−^ stock reactor, sequenced to approximately the same depth, contained bacteria from nine different bacterial phyla (Kantor et al., 2015). The draw and fill mode of operation of the solids reactor at the time of analysis should have favored retention of slow‐growing cells relative to the continuous flow mode of operation of the solids‐free reactor, which could have led to increased diversity in the solids reactor. However, this effect would have been countered by biofilm formation in the reactor without solids, which likely prevented washout of slow‐growing species, potentially increasing diversity in the solids‐free reactor. The bacteria detected represent only a subset of all organisms present in the SCN^−^ stock reactor, given that organisms from 17 bacterial phyla have been detected across three experiments that were inoculated from that source (Figure S1: SCN^−^ stock reactor + the CN–SCN^−^ reactor + SCN^−^ two‐reactor time series; Kantor et al., 2015; R. S. Kantor, R. J. Huddy, I. Ramsunder, B. C. Thomas, S. Tringe, R. L., Hettich, S. T. L., Harrison, J. F. Banfield, in review). The finding of lower bacterial diversity in the solids reactor compared with the solids‐free SCN‐stock reactor expands on results of a previous study that used a 16S rRNA gene clone library (30 sequences) to suggest lowering of diversity in reactors operated with solids and in continuous culture (van Zyl et al., 2015).

Differing conditions in the solids bioreactor, including the mode of operation, SCN^−^ loading rate, absence of biofilm, increased shear stress, and lower pH (van Zyl et al., 2015), likely affected the community composition. Truepera_1, an organism not previously detected in other reactors, was relatively abundant in the solids reactor, and may have been selected for due to its ability to withstand shear stress. The other bacteria enriched in the solids reactor were different species or different strains of species present in the other reactors derived from the same inoculum. The solids reactor exhibited lower diversity than any solids‐free reactor at the strain as well as the phylum level (Figure S1). Performance of the solids reactor over an extended duration achieved a sustained SCN^−^ degradation rate of 56 mg/L/h and was similar to the biofilm‐based communities in the solids‐free reactors, in terms of SCN^−^ degradation rates achieved relative to SCN^−^ loading. The solids reactor also exhibited short periods of compromised degradation in response to perturbation of preferred operating conditions over the experimental period (van Zyl et al., 2015). This may have been a consequence of decreased species diversity in the solids reactor relative to the solids‐free reactors and the presence of only one organism capable of SCN^−^ degradation.

Organisms that can respire aerobically dominated the solids reactor community (Table S1), as was expected since the reactor was well aerated and the biofilm was not present (van Zyl et al., 2015). A moderately abundant *Thiobacillus* in the solids reactor possessed the genes for SCN^−^ degradation (Figure 1), whereas in the solids‐free reactors, SCN^−^‐degrading thiobacilli were the dominant organisms (Kantor et al., 2015; R. S. Kantor, R. J. Huddy, I. Ramsunder, B. C. Thomas, S. Tringe, R. L., Hettich, S. T. L., Harrison, J. F. Banfield, in review). The comparatively lower relative abundance of *Thiobacillus* explains the reduced resilience of the solids reactor system to perturbation in terms of SCN^−^ degradation reported by van Zyl et al. (2015). Despite the differences between this reactor and the solids‐free reactors, several organisms in the solids bioreactor harbored genes for denitrification and sulfur oxidation (Table S1), key steps in the remediation of thiocyanate from wastewater.

We reconstructed genomes for 40 bacteria present in the solids reactor but only six of these were genomically sampled from bioreactors operated without solids (Figure 2). Thus, this genome‐resolved metagenomic analysis of the solids reactor expanded knowledge regarding organisms present in ASTER^™^ microbial consortium and increased available information about their metabolic potential.

## CONFLICT OF INTEREST

None declared.
