# Supplementary material for: Genome‐resolved metagenomics of a bioremediation system for degradation of thiocyanate in mine water containing suspended solid tailings
Source: Microbiologyopen. 2017 Feb 19;6(3):e00446. doi: 10.1002/mbo3.446 (PMC5458468; doi:10.1002/mbo3.446)
Supplement: Supplementary file 2 [file MBO3-6-na-s002.pdf]

									Carbon fixation		Aerobic respiration										SCN^- hydrolase	Ammonia oxidation		NO_3^- reduction		NO_2^- reduction		NO reduction		N_2O reduction		S^{2-} oxidation			H_2S oxidation		SO_3^{2-} oxidation	
**Genome Bin**	**Phylum/Class**	**Solids 1 coverage**	**Solids 2 coverage**	**Bin length (Mbp)**	**GC%**	**Number of scaffolds**	**Longest scaffold (bp)**	**Single copy genes (total: 51)**	*rbc* - Form I	*rbc* - Form II	*coxA*	*coxB*	*ccoN*	*ccoO*	*ccoP*	*cyoA*	*cyoD*	*cyoE*	*cydA*	*cydB*	*scnABC*	*amoC*	*hao*	*narG*	*narH*	*nirK*	*nirS*	*norB*	*norC*	*nosD*	*nosZ*	*soxC*	*dsrA*	*dsrB*	*fcc*	*sqr*	*apr*	*atpS*
solids_Sphingobacteriales_2	Bacteroidetes	739	559	3.560973	40.11	254	71409	49					1	1				1						1	1	2		1	2	1	1							
solids_Rhodanobacter_3	Gammaproteobacteria	174	98	3.459585	68.74	123	139761	48			1	1	1		1			1	1	1				1	1	2		4	1	1						1		
solids_Burkholderiales_1	Betaproteobacteria	132	33	3.556883	65.94	27	790171	48	1		1	1	2	1	1			1					1			1	1	1	1			2			3			
solids_Rhodanobacter_2	Gammaproteobacteria	115	346	2.95749	68	332	48323	27			1	1	3	1	1			1	1	1						2		4	1									
solids_Truepera_1	Deinococcus-Thermus	105	77	1.220355	66.58	327	45361	46			1	1						1								1												
solids_Thiobacillus_2	Betaproteobacteria	101	108	0.671397	62.87	176	25668	39													3			1						1	1				1	2	1	
solids_Actinobacteria_1	Actinobacteria	93	230	3.443405	68.93	71	208764	50			2							1						1	1											1		
solids_Burkholderiales_2	Betaproteobacteria	82	4.9	2.272635	65.43	417	102603	32	1		1		1		1			3						2	1		1			1	1	1			1			
solids_Sphingobacteriales_1	Bacteroidetes	80	35	3.293488	39.22	18	591082	50					2	1	1			1						1	1	1			1	1	1							
solids_Microbacterium_1	Actinobacteria	56	25	3.028969	69.03	17	468500	51			1	1						1	1	1				1	1	1												
solids_Rhodanobacter_1	Gammaproteobacteria	49	144	2.762428	68.02	322	59675	30				1	3	1	1			1	2	1						2		2		1								
solids_Rhodanobacter_6	Gammaproteobacteria	47	28	0.749597	68.15	209	44573	9																				2	1									
solids_Sphingopyxis_1	Alphaproteobacteria	39	1.9	0.670628	65.34	306	13764	38			1	1	1		1	1		1	1	1																		
solids_Rhodanobacter_5	Gammaproteobacteria	36	13	3.602337	66.95	175	120909	37			1	1	1		1			1	1	1								1								1		
solids_Rhodanobacter_4	Gammaproteobacteria	30	29	3.432851	66.33	89	285871	23			1	1	2	1	1			1	1	1						2		1	1							1		
solids_Nitrosospira_1	Betaproteobacteria	28	30	3.080869	54.42	44	307813	51	1		1	1			1			1				1	1			1		1	1							2		
solids_Sphingobacteriales_4	Bacteroidetes	25	7.3	4.668254	38.37	70	293464	51					1		1			1						1	1	1				1	1							
solids_Brevundimonas_1	Alphaproteobacteria	23	1.6	1.485446	67.83	670	26323	45			1	1						1	1																	1		
solids_Castellaniella_1	Betaproteobacteria	23	77	3.708385	66.07	51	574638	51			1	1	3	2	1	1	1	1	1					1	1	2		2	1	1	1					1		
solids_Stenotrophomonas_1	Gammaproteobacteria	21	1.2	3.463406	69.59	89	203045	50				1	1		1	1	1	1	1	1				1	1	1		1										
solids_Burkholderiales_3	Betaproteobacteria	16	4.6	1.404795	65.94	575	69432	18				1			1			1	1																			
solids_Alphaproteobacteria_1	Alphaproteobacteria	14	39	3.200314	61.99	66	253970	49			1	1	2	1	1			1	1	1								1										
solids_Rhizobiales_1	Alphaproteobacteria	14	16	4.689115	63.33	69	485647	51			2	1	1		1			1	1	2				1	1	3		1	1			1						
solids_Sphingobacteriales_3	Bacteroidetes	12	18	5.329888	42.32	116	223840	51					2	1	1			1						1	1	2		1		1	1							
solids_Thiobacillus_1	Betaproteobacteria	11	16	1.935166	64.27	229	53321	44	1	1	1	1	2		3			1	1	1				1				1		1	1		2	1	1			
solids_Actinobacteria_3	Actinobacteria	9.7	12	3.028281	70.55	224	59440	50			2	1							1					1	1													
solids_Xanthomonadales_1	Gammaproteobacteria	9.5	13	3.327705	65.7	24	411702	45			1	1	2	2	1	1	1	1	1					1	1			1										1
solids_Xanthomonadales_2	Gammaproteobacteria	8.4	3.7	1.825173	68.58	191	34686	18				1	1	1	1			1									1	1										
solids_Lysobacter_1	Gammaproteobacteria	7.9	3.4	0.432826	68.46	156	16506	26																1	1	1												
solids_Afipia_1	Alphaproteobacteria	7.5	19	2.809209	61.18	27	315568	51					1	1										1	1	2						1						
solids_Sphingopyxis_2	Alphaproteobacteria	7.3	2.4	1.436326	66.44	641	11619	30									1											1										
solids_Gemmatimonadetes_1	Gemmatimonadetes	6.3		3.096685	65.71	623	29115	47			1	1	2	1	1									1		1		1		1	1							
solids_Cytophagia_1	Bacteroidetes	5.2	8.5	1.403249	34.1	138	53453	49																														
solids_Micrococcales_1	Actinobacteria	5	16	1.843805	68.98	1001	8486	24							1									1														
solids_Bacteroidetes_1	Bacteroidetes	4.6	12	3.159377	37.08	83	248971	46					1	1	2			1								1				1	1							
solids_Flavobacteriales_1	Bacteroidetes	4.2	2.7	1.307045	39.03	814	8954	11						1																								
solids_Rhizobiales_2	Alphaproteobacteria	2.9	9.1	2.693058	66.69	167	56037	19			1	1	2	1	1			1	1	1						1												
solids_Flavobacteriia_1	Bacteroidetes	2.1	53	3.160681	39.49	20	531725	49					2					1																				
solids_Actinobacteria_2	Actinobacteria	1.9	14	5.135461	69.54	86	293326	45				1			1			1	1	1				1	1													
solids_Chryseobacterium_1	Bacteroidetes	1.6	40	2.280943	39.38	23	311201	50					2	2	1											1			1	1	1							

Table S1: Genome statistics and metabolic potential for the 40 bacterial genome bins in the solids bioreactor dataset (http://ggkbase.berkeley.edu/scnpilot_solids_dereplicated/organisms), ordered by normalized coverage in the solids 1 sample. The numbers represent the count of each indicated gene.
