# Supplementary material for: Genome‐resolved metagenomics of a bioremediation system for degradation of thiocyanate in mine water containing suspended solid tailings
Source: Microbiologyopen. 2017 Feb 19;6(3):e00446. doi: 10.1002/mbo3.446 (PMC5458468; doi:10.1002/mbo3.446)
Supplement: Supplementary file 3 [file MBO3-6-na-s003.docx]

Figure S1: Phylogenetic placement of genomes using a maximum-likelihood tree based on concatenated alignments of 16 ribosomal proteins. Colors of the tip labels indicate the following: red for the solids reactor (http://ggkbase.berkeley.edu/scnpilot_solids_dereplicated/organisms) (this study), blue for the SCN^-^ stock reactor (http://ggkbase.berkeley.edu/SCN-stock/organisms) (Kantor *et al.,* 2015), purple for the CN-SCN reactor (<http://ggkbase.berkeley.edu/CN-SCN/organisms>) (Kantor *et al.,* 2015), and green for the SCN^-^ two-reactor time series (http://ggkbase.berkeley.edu/scnpilot-dereplicated/organisms) (Kantor *et al.,* in prep). Some genomes from these datasets are not included in the tree due to incomplete ribosomal protein sequences. Phyla that include genomes from the SCN^-^ bioreactor datasets are labeled.

Table S1: Genome statistics and metabolic potential for the 40 bacterial genome bins in the solids bioreactor dataset (http://ggkbase.berkeley.edu/scnpilot_solids_dereplicated/organisms), ordered by normalized coverage in the solids 1 sample. The numbers represent the count of each indicated gene.
